# Structural diversification of vitamin D using microbial biotransformations

**DOI:** 10.1007/s00253-024-13244-w

**Published:** 2024-07-06

**Authors:** Mario García-Domínguez, Ignacio Gutiérrez-del-Río, Claudio J. Villar, Anabel Perez-Gomez, Ignacio Sancho-Martinez, Felipe Lombó

**Affiliations:** 1 Research Group BIONUC (Biotechnology of Nutraceuticals and Bioactive Compounds), Departamento de Biología Funcional, Principality of Asturias, Área de Microbiología, Universidad de Oviedo, Oviedo, Spain; 2 IUOPA (Instituto Universitario de Oncología del Principado de Asturias), Oviedo, Spain; 3 ISPA (Instituto de Investigación Sanitaria del Principado de Asturias), Oviedo, Spain; 4 Department of Drug Discovery, FAES Farma, Leioa, Bizkaia Spain

**Keywords:** Cholecalciferol, Ergocalciferol, Calcitriol, Hydroxylase, Biotransformation

## Abstract

**Abstract:**

Vitamin D deficiencies are linked to multiple human diseases. Optimizing its synthesis, physicochemical properties, and delivery systems while minimizing side effects is of clinical relevance and is of great medical and industrial interest. Biotechnological techniques may render new modified forms of vitamin D that may exhibit improved absorption, stability, or targeted physiological effects. Novel modified vitamin D derivatives hold promise for developing future therapeutic approaches and addressing specific health concerns related to vitamin D deficiency or impaired metabolism, such as avoiding hypercalcemic effects. Identifying and engineering key enzymes and biosynthetic pathways involved, as well as developing efficient cultures, are therefore of outmost importance and subject of intense research. Moreover, we elaborate on the critical role that microbial bioconversions might play in the *a la carte* design, synthesis, and production of novel, more efficient, and safer forms of vitamin D and its analogs. In summary, the novelty of this work resides in the detailed description of the physiological, medical, biochemical, and epidemiological aspects of vitamin D supplementation and the steps towards the enhanced and simplified industrial production of this family of bioactives relying on microbial enzymes.

**Key points:**

• *Liver or kidney pathologies may hamper vitamin D biosynthesis*

• *Actinomycetes are able to carry out 1α- or 25-hydroxylation on vitamin D precursors*

## Introduction

**Fig. 1:**
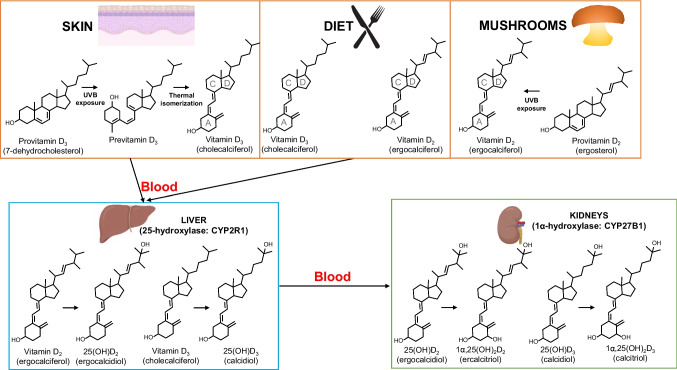
Biosynthetic pathways for acquisition of vitamin D_3_ (via solar irradiation in the skin or from diet) and vitamin D_2_ (from diet), as well as liver and kidney biotransformations (hydroxylations) taking place on both types of vitamin D

The term vitamin D refers to a group of compounds with similar chemical structures and properties, including dietary vitamin D_2_ (ergocalciferol, fungal origin) and vitamin D_3_ (cholecalciferol, animal origin). Both types of vitamin D have a basic steroidal structure, which means they share a common backbone derived from ergosterol (provitamin D_2_, in fungi) or 7-dehydrocholesterol (provitamin D_3_, in human skin) (Fig. 1) (Borel et al. 2015). In human plasma, the primary vitamin D metabolite is the pro-hormone known as 25-hydroxy-vitamin D (calcidiol). This monohydroxy pro-hormone is synthesized in the liver through the hydroxylation process of either the precursor vitamin D_2_ (ergocalciferol, which is produced in plants and fungi following exposure to UVB radiation on ergosterol) or vitamin D_3_ (cholecalciferol, formed in the skin under the influence of UVB radiation acting on a cholesterol derivative called 7-dehydrocholesterol) (Fig. 1) (Borel et al. 2015). It is worth noting that the biologically active form of this vitamin is 1α,25-dihydroxy-vitamin D (calcitriol), which needs a biosynthetic step in the kidney and is typically found in plasma at concentrations approximately 1000 times lower than its precursor (Lips 2007).

Dietary, physiological, and societal factors may reduce the plasma concentration of vitamin D precursors, and may therefore contribute to diverse organ and tissue malfunctions, such as osteoporosis, rickets, fatigue, depression, or higher susceptibility to diverse infections. A practical solution to this deficiency is oral supplementation with vitamin D precursors (vitamin D_3_, vitamin D_2_) or their mono- or dihydroxylated forms (calcidiol, ergocalcidiol, calcitriol, ergocalcitriol), depending on specific liver (where the 25-hydroxylation must take place) or kidney (where the 1α-hydroxylation must take place) pathologies present in the treated individual (Gallagher and Rosen 2023; Płudowski et al. 2023; Sizar et al. 2023).

Beyond chemical synthesis processes used widely in industry for the production of vitamin D, which usually start from a natural backbone precursor (cholesterol, ergosterol, or 7-dehydrocholesterol), some microorganisms (e.g., actinomycetes and *Bacillus* species) are able to carry out these conversions or the generation of vitamin D derivatives by using their broad enzymatic machinery, such as cytochromes P_450_. Among these industrially interested microbial enzymes, 1α-hydroxylases and 25-hydroxylases have been identified and studied from diverse bacterial species (Abdulmughni et al. 2017; Ang et al. 2018; Hayashi et al. 2010; Schmitz et al. 2021). The biological importance of vitamin D and the industrial alternatives developed for its pharmaceutical supply will be described along the next chapters.

The physiological (calcium and phosphate homeostasis, immune system function, cardiovascular health), medical (presence of liver or kidney malfunction, osteoporosis, pregnancy, breastfeeding, pharmacological management), biochemical (VDR-dependent and independent responses), and epidemiological (social, gender, and geographical factors) reasons for maintaining a high research investment in the pharmaceutical production (chemical synthesis, combinatorial biosynthesis) of vitamin D compounds with improved bioactivities (cardioprotection, anti-inflammatory, neuroprotection, etc.) and lesser side effects (such as hypercalcemia) are described in detail in the following sections.

## Epidemiology of vitamin D deficiency

Vitamin D deficiency is defined by serum levels of certain vitamin forms falling below a certain threshold, typically considered to be plasma levels lower than 20 ng/mL of 25-hydroxy-vitamin D (Amrein 2020). The prevalence of this condition has been reported worldwide, with rates reaching more than 20% in India (Cashman et al. 2016), 13% in Europe (Cashman et al. 2016), 7.4% in Canada (Sarafin et al. 2015), and 5.9% in the USA (Schleicher et al. 2016). Ethnicity plays a role, particularly in populations with darker skin (e.g., in Africa, Oceania, and certain areas of South Asia), where reduced sunlight exposure due to melanin inhibits vitamin D_3_ synthesis (Merchant et al. 2018). Independently of the country, some populations are prone to more frequent vitamin D deficiency, due to the lack of adequate sun exposure, such as indoor-centric professionals, or lifestyle choices as people wearing veils and other protective clothing (Al-Yatama et al. 2019; Gallagher and Rosen 2023; Sowah et al. 2017). Among broader factors, dietary habits significantly influence vitamin D status. Many populations exhibit dietary patterns low in vitamin D–rich foods such as egg yolks, fatty fish (salmon, sardines, herring, tuna, cod), liver, or fortified products like butter, milk, and yogurt (Lamberg-Allardt 2006). The adoption of national policies on vitamin D supplementation, like in the Scandinavian countries, has reduced its deficiency to less than 1% of the population (Gallagher and Rosen 2023).

Another critical factor associated to vitamin D levels is age. For example, in the case of lactating babies, excessive breastfeeding can lead to vitamin D deficiency, as breast milk is generally low in vitamin D (O’Callaghan et al. 2020). Conversely, elderly individuals face an elevated risk of pathologies stemming from vitamin D deficiency due to factors like diminished skin synthesis of vitamin D (owing to less sun exposure) and metabolic changes, including reduced kidney formation of 1α,25-dihydroxy-vitamin D, or decreased intestinal calcium absorption due to a decline in vitamin D receptor (VDR) intestinal expression (Gallagher 2013).

Both gender-specific and socioeconomic statuses have emerged as parameters potentially affecting vitamin D levels in humans (Sutherland et al. 2021). Gender-wise, recent studies highlight a higher prevalence of vitamin D deficiency in males, possibly attributed to a more sedentary lifestyle, reducing sunlight exposure time (Ravelo et al. 2022). Pregnant women may also encounter vitamin D deficiency due to heightened nutritional demands not being met (Mithal and Kalra 2014). Smokers often require vitamin D supplements as serum levels are significantly lower compared to non-smokers, likely due to a reduction of parathyroid hormone (a key factor enhancing renal conversion of 25-dihydroxy-vitamin D to 1,25-dihydroxy-vitamin D) caused by smoke components (Yang et al. 2021).

Last, individuals with certain medical conditions are notably susceptible, or even directly related, to vitamin D deficiency. These conditions include liver diseases such as non-alcoholic fatty liver disease and hepatitis C, kidney diseases like chronic kidney disease, and gastrointestinal disorders such as cystic fibrosis, celiac disease, and inflammatory bowel disease. In the context of disease, particularly chronic kidney disease, it is crucial to understand the interplay between vitamin D, the kidneys, and parathyroid hormone (PTH) which constitutes an endocrine loop instrumental in calcium homeostasis. Vitamin D, primarily in the form of calcitriol (1α,25-dihydroxy-vitamin D), facilitates calcium absorption in the gut. When vitamin D levels are insufficient, the parathyroid glands respond by secreting increased amounts of PTH. This hormone, in turn, stimulates the conversion of calcidiol (25-hydroxy-vitamin D) to calcitriol (1α,25-dihydroxy-vitamin D) in the kidneys, enhancing calcium reabsorption and phosphate excretion. Moreover, PTH induces the release of calcium from bone to restore serum calcium levels. This endocrine loop showcases the intricate relationship between vitamin D, kidney function, and PTH secretion in maintaining calcium and phosphate balance within the body. Thus, calcitriol supplementation is often considered for these individuals to mitigate the deficiency (Iruzubieta et al. 2014; Margulies et al. 2015; Williams et al. 2009). The deficiency risk is further heightened in individuals with higher melanin content in their skin, as melanin impedes the skin’s ability to produce the vitamin D_3_ precursor (Margulies et al. 2015).

## Clinical aspects of vitamin D deficiency

Most individuals with vitamin D deficiency are asymptomatic (Nadeem et al. 2018). However, individuals with a moderate vitamin D deficiency can experience symptoms including intense bone pain, fatigue, myalgias, arthralgias, and weakness (Sizar et al. 2023), increased susceptibility to microbial infections (Chalmers et al. 2013; Ryz et al. 2015), hair loss (Saini et Mysore 2021), depression, and mood changes (Lavigne et al. 2023; Guzek et al. 2021). In pregnant women, reduced vitamin D levels are associated to higher risk of cesarean delivery, gestational diabetes, pre-eclampsia, and preterm delivery (Płudowski et al. 2023). In the elderly population, prolonged vitamin D deficiency causes secondary hyperparathyroidism, which in turn causes severe loss of bone mass, leading first to osteomalacia (bone softening) (Bhan et al. 2010) and subsequently to osteoporosis (formation of large spaces on the bone structure) (Hill and Aspray 2017), therefore increasing the risk of suffering osteoporotic fractures (Looker 2013). In children, prolonged deficiency induces osteomalacia and rickets, a pathology characterized by symptoms such as leg deformity, frontal bossing, delayed tooth eruption, and bone pain (Chanchlani et al. 2020). In the EU, 25-hydroxy vitamin D serum values below 20 ng/mL (50 nmol/L) indicate deficiency status, and values between 20 and 30 ng/mL are considered suboptimal status. Serum concentrations over 100 ng/mL (250 nmol/L) are considered at risk of intoxication (Płudowski et al. 2023).

A large part of vitamin D needs are covered with exposure to sunlight. Nevertheless, it is impractical to suggest a one-size-fits-all duration of sun exposure that would be adequate to fulfill the essential annual vitamin D needs for everyone, as several factors need to be considered, such as age, physical characteristics, weather conditions, and season (Chang and Lee 2019; Dominguez et al. 2021). Vitamin D deficiency treatment encompasses dietary modifications (such as dried mushrooms, cod liver oil, oily fish), supplementation, and enhanced sun exposure (Płudowski et al. 2023). Incorporating diverse foods like mushrooms, fatty fish, and eggs can fulfill daily vitamin D requirements (Pilz et al. 2018; Dominguez et al. 2021).

In cases where sun exposure and feeding habits are not sufficient, vitamin D supplementation is highly recommended. These supplements are available in numerous forms, including vitamin D_2_ and vitamin D_3_. The amount of vitamin D required to treat its deficiency depends largely on the degree of the deficiency and underlying risk factors. An initial supplementation for 8 weeks with vitamin D_3_ at either 6000 IU daily (10,000 IU for high-risk adults, 2000 IU for children) or 50,000 IU weekly is usually administered. And then, a daily maintenance dose of 1000 (for children) to 2000 IU is recommended (3000 to 6000 IU for high-risk adults). In people where the deficiency persists despite treatment with vitamin D_2_ or D_3_, calcitriol may be administered, specially when a chronic kidney disease is responsible for that deficiency. Calcidiol can be considered in patients with fat malabsorption or severe liver disease (Gallagher and Rosen 2023; Sizar et al. 2023). In all cases, medical supervision is needed before starting any treatment, including a blood test to determine plasma vitamin D levels to confirm the effectiveness of the treatment applied (Reid and Bolland 2019).

## Bioactivities of vitamin D

Vitamin D (vitamin D_2_ and D_3_) and their mono- and dihydroxylated derivatives are a family of lipophilic molecules that play a fundamental role in developing and maintaining human health. Beyond its well-established role in calcium homeostasis and bone physiology mentioned above (Heaney 2011a, b; Laird et al. 2010; Tan et al. 2018), emerging research has unraveled the influence of vitamin D on other physiological conditions, such as immune (phagocytosis, antimicrobial peptide expression), nervous (mood), or cardiovascular (hypertension) (Zmijewski 2019).

Vitamin D exerts its biological actions through a very complex mechanism involving its activation, which takes place primarily in the skin upon exposure to UVB radiation from sunlight. Alternatively, vitamin D can also be obtained through dietary sources, such as fatty fish or mushrooms (Balachandar et al. 2021). The canonical genomic activity of vitamin D occurs upon binding of the 1α,25-dihydroxy-vitamin D to the vitamin D receptor. VDR is widely distributed throughout the body, with high expression levels in tissues such as the intestines, bones, kidneys, and immune cells (Wang et al. 2012). Upon activation, VDR binds to the retinoic acid receptor (RXR) to exert its activities as a multiprotein transcription factor complex binding to specific DNA sequences (called vitamin D response elements or VDREs), acting as a transcription factor with a very high affinity for the vitamin (0.1 nM) (Carlberg 2022). These sequences are located in the promoter regions of target genes, such as those involved in calcium metabolism (see section “Epidemiology of vitamin D deficiency” above) (Pike et al. 2017; Costa et al. 2019; Carlberg 2022). Interestingly, more than 900 polymorphisms are known for the VDR transcription factor gene which might lead to differential VDR activities and, in certain cases, link to disease. For example, polymorphisms, such as rs2228570, affecting exon 2, generate a shorter version of the transcription factor (424 aa instead of wild-type 427 aa) with augmented transcriptional activity, which has been associated to higher risk of systemic lupus erythematosus, type 1 diabetes, and Hashimoto’s thyroiditis. The rs1544410 and rs7975232 polymorphisms are variants with the same number of amino acids, 427, but lower expression levels due to mRNA instability. The former has been associated to higher risk of rheumatoid arthritis and systemic lupus erythematosus, and the latter to higher risk of systemic lupus erythematosus, multiple sclerosis, and vitiligo. The rs731236 polymorphism shows an altered splicing, and its variants are associated to multiple sclerosis risk. The rs739837 polymorphism at the 3’ end is associated to higher type 2 diabetes risk. In a similar way, other polymorphisms are associated with a higher risk of metabolic syndrome (rs7975232) or hypertension (rs2228750) (Agliardi et al. 2023; Awasthi et al. 2023; Fronczek et al. 2023).

As mentioned above, vitamin D plays a pivotal role in maintaining calcium and phosphate homeostasis in the body, with a network of hormones, including FGF23 from bone and parathyroid hormone, collaboratively regulating various physiological processes in the intestine, bones, and kidneys (Mace et al. 2020; Shaker and Deftos 2000). FGF23, produced by osteocytes and osteoblasts in bones, serves a dual role. It promotes the excretion of calcium and phosphate in urine, and tightly controls the synthesis and degradation of 1α,25-dihydroxy-vitamin D in the kidneys. Additionally, in the parathyroid gland, FGF23 inhibits the production of parathyroid hormone by binding to the FGFR1c and alpha Klotho receptor:coreceptor complex. On the other hand, parathyroid hormone influences calcium dynamics by mobilizing it from bone tissue via osteoclasts, facilitating calcium reabsorption in the kidneys, and enhancing phosphate renal excretion. Parathyroid hormone also triggers the biosynthesis of 1α,25-dihydroxy-vitamin D in the kidneys. However, this inhibitory effect of FGF23 on parathyroid hormone is not observed when calcium levels are low (Mace et al. 2020).

In bone, the synthesis of FGF23 is stimulated by elevated plasma phosphate levels. Moreover, the binding of parathyroid hormone to its FGFR receptor in bone cells activates the transcription of FGF23. Similarly, 1α,25-dihydroxy-vitamin D, through its VDR receptor, acts as a transcriptional activator for FGF23. Subsequently, FGF23 exerts its activities upon binding to FGFR1c and alpha Klotho heterodimers within the kidney’s distal tubules. Binding of FGF23 to its receptor FGFR1c and the coreceptor alpha Klotho ultimately leads to the inhibition of phosphate reabsorption by degrading the sodium-phosphate cotransporter NaPi2a and the induction of calcium reabsorption by overexpressing the calcium channel TRPV5 (Mace et al. 2020). Furthermore, FGF23 suppresses the activity of 1α-hydroxylase in the proximal renal tube, responsible for converting 25-dihydroxy-vitamin D into 1α,25-dihydroxy-vitamin D. Simultaneously, FGF23 enhances the activity of renal 24-hydroxylase, which is involved in the catabolism of 25-dihydroxy vitamin D (Mace et al. 2020).

Notwithstanding, the intricate interplay among FGF23, parathyroid hormone, and 1α,25-dihydroxy-vitamin D holds significance in the context of a pathological condition known as mineral and bone disorder, which occurs in individuals with chronic kidney disease (CKD-MBD). In these patients, a cascade of effects leads to osteoporosis and soft tissue calcifications in the arteries and heart valves. This is attributed to an increase in plasma FGF23 concentration, possibly due to kidney filtration failure, and a deficiency in the Klotho coreceptor, resulting in reduced renal biosynthesis of 1α,25-dihydroxy-vitamin D. Consequently, low plasma levels of 1α,25-dihydroxy-vitamin D induce secondary hyperparathyroidism, as circulating phosphate cannot be excreted normally in the tubules (Mace et al. 2020). Interestingly, a pharmacological approach involving the use of a vitamin D analog called doxercalciferol (1α-hydroxy-vitamin D_2_) has been approved for the management of secondary hyperparathyroidism in CKD; notably, this drug only requires the 25-hydroxylation step, which occurs in the liver and remains functional in renal patients. Doxercalciferol effectively reduces parathyroid hormone levels and restores normal bone physiology (Park et al. 2014).

At the mitochondria level, the organelle where the activation hydroxylation steps take place for 1α,25-dihydroxy-vitamin D, this vitamin (bound to VDR in mitochondria cytoplasm) can regulate some mitochondrial genes involved in oxidative phosphorylation, defense against reactive oxygen species, and fusion/fission processes (Zmijewski 2019).

Persistently low serum levels of vitamin D are implicated in serious health conditions. For instance, such deficiencies contribute to rickets, evidenced by skeletal anomalies in children (Castano et al. 2022; Mungai et al. 2021), and to osteoporosis, with its accompanying bone fragility and pain in adults (Minisola et al. 2020). The significance of vitamin D during pregnancy is equally critical, showing an increase in 1α,25-dihydroxy-vitamin D serum levels early in pregnancy regardless of PTH levels, as it fosters the development of the fetal skeletal system (Castano et al. 2022; Gallo et al. 2020). Calcidiol deficiencies during pregnancy have been associated with an increased prevalence of preeclampsia, low birth weight, and neonatal hypocalcemia (Mulligan et al. 2010). Once a woman gives birth, 1α,25-dihydroxy-vitamin D levels return to preconception values, but mothers who breastfeed their babies still have a high calcidiol requirement (Castano et al. 2022).

Recent studies have expanded our understanding of vitamin D’s role, highlighting its regulatory effects on the immune system. Since cells of the immune system can express the vitamin D receptor, they respond in a coherent fashion when exposed to 1α,25-dihydroxy-vitamin D. Vitamin D exhibits immunomodulatory properties, enhancing our defense against microbial agents through the stimulation of antimicrobial peptides like defensins and cathelicidins (Taha et al. 2021; Youssef et al. 2011), and reducing the incidence of autoimmune disorders (Sîrbe et al. 2022). Adequate levels of vitamin D correlate with reduced susceptibility to severe respiratory infections, including SARS-CoV-2 (Torres et al. 2022). The available data suggests that maintaining adequate serum 25-hydroxy-vitamin D levels in patients with SARS-CoV-2 infection may significantly reduce the risk of acute respiratory distress syndrome (ARDS) and severe COVID-19, with possible beneficial effects on the need for mechanical ventilation and/or intensive care unit (ICU) admission, as well as deaths in the course of the disease (Quesada-Gomez et al. 2022; Bouillon and Quesada-Gomez 2021). In addition, vitamin D shows promise in improving outcomes in autoimmune diseases such as rheumatoid arthritis (Harrison et al. 2020), amyotrophic lateral sclerosis (Gianforcaro and Hamadeh 2014), multiple sclerosis (Sintzel et al. 2018), and type I diabetes (Chakhtoura and Azar 2013).

The cardiovascular system also reaps the benefits of vitamin D, with studies indicating a potential reduction in risks associated with hypertension, coronary artery disease, and heart failure (de la Guía-Galipienso et al. 2021; Latic and Erben 2020). More specifically, low plasma levels of vitamin D are associated with vascular damage and also play an important role in myocardial status (Assalin et al. 2013; Tuñón et al. 2016). Although the negative association between vitamin D deficiency and cardiovascular disease has been described in a multitude of studies in animal models, clinical trials have failed to demonstrate the benefit of vitamin D supplementation on cardiovascular health (Tuñón et al. 2016). Additionally, there is an inverse relationship between vitamin D and the risk of developing type 2 diabetes, as it appears to enhance insulin sensitivity (Lips et al. 2017). The vitamin’s role in cancer prevention is noteworthy, particularly concerning colorectal cancer (Javed et al. 2020), where it regulates cell proliferation, promotes apoptosis, and inhibits angiogenesis (Carlberg and Muñoz 2022). These protective effects extend to its anti-inflammatory and antiproliferative activities in various cancer types (Jeon and Shin 2018).

In addition, vitamin D deficiencies have been associated with an increased risk of mental health disorders, including depression (Menon et al. 2020) and dementia (Roy et al. 2021). However, it is crucial to note that there is an upper safe limit for vitamin D intake, typically between 4000 and 10,000 IU daily (Grygorieva et al. 2023). Exceeding this threshold can result in hypercalcemia and associated symptoms such as nausea and confusion, as well as the potential for renal deposits, bone demineralization, and an elevated fracture risk (Burt et al. 2019; Kaufmann et al. 2021). Although excessive vitamin D has not shown adverse cardiovascular effects (Zittermann et al. 2021), maintaining its balance is imperative for preventing elevated parathyroid hormone levels and associated fracture risks (Mendes et al. 2020).

Apart from all the genomic activities of vitamin D, directly linked to its binding to VDR transcriptional activator, vitamin D also possesses diverse non-genomic activities of importance for cell homeostasis, associated to its binding to other types of receptors. These include endocytosis of vitamin D (bound to VDBP) mediated by megalin in the muscle, mammary gland, placenta, colon, and skin. Also, in myocytes, vitamin D stimulates the calcium current, in a way dependent on protein kinase C (PKC), contributing to these cells’ contractility. Finally, vitamin D is able to protect against oxidative stress due to UVB radiation as well, giving rise to inactive side products such as 5,7-dienes and to an increase in the tumor suppressor protein p53.

## Sources of vitamin D

Sunlight exposure is the most significant source of vitamin D_3_, usually accounting for around 80% of total vitamin D entering the body (Jeon and Shin 2018). When the skin is exposed to UVB radiation, a provitamin D_3_ molecule (7-dehydrocholesterol) present in this tissue undergoes a chemical reaction and is converted into previtamin D_3_ (Fig. 1). Subsequently, the skin temperature originates the transformation of this previtamin D_3_ into vitamin D_3_ or cholecalciferol (Fig. 1). The plasma levels of vitamin D_3_ depend on factors such as weather conditions, latitude, skin pigmentation, or the use of sunscreen lotions (Oskarsson et al. 2022; Knuschke 2021). As an example, sun radiation exposure over the face, hands, and arms for 15 min in people with low melanin content skin may produce about 200 to 600 IU of cholecalciferol. Although a daily dose of 600 to 800 IU is considered adequate for a healthy state, the extra amount can be obtained also from some foods (Chang and Lee 2019; Dominguez et al. 2021).

Normal vitamin D_2_ (from fungal sources) or D_3_ (from animal sources) daily intake shows a broad range from 400 to 10,000 IU, although the recommended values may vary depending on age and some physiological conditions. For example, the tolerable upper intake limits are 1000 IU (normal supplementation 400–600 IU per day) for infants under 12 months, 2000 IU (normal supplementation 600–1000 IU per day) for children under 10 years, 4000 IU (normal supplementation 1000–2000 IU per day) for adolescents under 18 years, adults with normal body weight, and pregnant women, and 10,000 IU for adults with obesity. In the case of preterm neonates, 400–1000 IU per day is recommended (Sizar et al. 2023; Płudowski et al. 2023). The main sources that provide vitamin D in the human diet are cod liver oil (1360 IU per tablespoon); salmon and other fatty fishes such as tuna or trout (670 IU per 100 g); mushrooms exposed to sunlight (430 IU per 100 g), which are a rich source of dietary vitamin D_2_ for vegetarians and vegans; eggs (51 IU per unit, but more if hens are exposed to controlled UVB radiation), or cheese (14 IU per 100 g) (Bartolucci et al. 2011; Chang and Lee 2019; Dominguez et al. 2021; Kühn et al. 2014; Leung and Cheung 2021; Lu et al. 2007). In some cases, vitamin D_2_ foods that naturally do not contain significant amounts of this vitamin, such as plant-based dairy drinks or breakfast cereals, are supplemented with it (Itkonen et al. 2016).

In clinical practice, the pharmacological management of vitamin D deficiency entails the administration of two principal forms of the vitamin: ergocalciferol (vitamin D_2_) and cholecalciferol (vitamin D_3_). Ergocalciferol is synthesized through the ultraviolet radiation of ergosterol extracted from the cell membranes of fungi and yeast, while cholecalciferol is derived from 7-dehydrocholesterol in lanolin, obtained from sheep’s wool, or fish liver oils. Cholecalciferol boasts a superior affinity for vitamin D binding proteins in plasma, leading to a more extended circulatory half-life, and greater efficacy in raising and maintaining serum 25-hydroxy-vitamin D concentrations, the principal circulating form of the vitamin, and a central indicator of vitamin D status.

Pharmaceutical preparations of vitamin D are available in various dosages for oral and intramuscular administration, facilitating personalized dosing regimens that can be adjusted based on the severity of the deficiency, the presence of comorbid conditions, and the individual’s response to therapy. The purity and concentration of pharmaceutical-grade vitamin D are rigorously controlled under stringent manufacturing standards to ensure efficacy and safety. Further sophistication in pharmaceutical vitamin D involves the use of active metabolites like calcitriol (1α,25-dihydroxy-cholecalciferol), the biologically active form of vitamin D, which bypasses the need for renal conversion that is a requisite for both D_2_ and D_3_ forms. This is particularly beneficial in patients with renal failure, where the conversion to the active form is compromised. Other analogs, such as calcidiol (25-hydroxy-cholecalciferol), provide a more direct elevation of the deficient 25-hydroxy-vitamin D levels with a faster onset of action, which is advantageous in acute deficiency states; in fact, several studies have compared the efficacy of calcidiol and cholecalciferol in increasing serum 25-hydroxy-vitamin D levels, showing that calcidiol is faster and more potent than cholecalciferol (about three times more potent in subjects with mild vitamin D deficiency) (Bouillon et al. 2023; Graeff-Armas et al. 2020). This rapid increase can be explained at the intestinal level. The intestinal absorption of calcidiol is close to 100% (due to its higher polarity) and it is rapidly absorbed by the intestinal cells and transported by the portal vein and therefore immediately accessible to the circulation; on the other hand, vitamin D is incorporated into the chylomicrons and absorbed by the lymphatic system, through which it enters the circulation. For this reason, calcifediol is an excellent alternative in the event of intestinal fat malabsorption, after bariatric surgery or in other conditions that compromise hydroxylation at the liver level (Bouillon and Quesada Gomez 2023). Although there are many clinical trials demonstrating the efficacy and safety of short-term calcidiol supplementation, its effects after long-term monthly administration have been studied less extensively. A recent clinical trial in postmenopausal women with vitamin D deficiency has shown that long-term treatment with monthly calcidiol in patients with vitamin D deficiency is effective and safe, generating stable and sustained 25-hydroxy-vitamin D levels that are not achieved if supplementation is stopped, which leads to a pronounced drop in 25-hydroxy-vitamin D levels (Pérez-Castrillón et al. 2023).

Recent advances have introduced novel delivery systems such as liposomal encapsulation, which have shown promise in enhancing the bioavailability of vitamin D. Liposomes are phospholipid vesicles that can encapsulate hydrophobic molecules like vitamin D, promoting increased absorption through the intestinal epithelium, and protection from degradation within the gastrointestinal tract. This technology represents a significant leap forward in the pharmacokinetics of vitamin D administration, potentially reducing the frequency of dosing and minimizing side effects.

## Synthesis and biosynthesis of vitamin D

Vitamin D backbone consists of three rings (labeled A, C, and D; Fig. 1) linked by an unsaturated aliphatic chain (Sirajudeen et al. 2019). The specific arrangement of atoms within these rings, mainly hydroxylation tailoring, gives vitamin D its characteristic shape (Campbell et al. 2010). Bound to ring D, there is an aliphatic side chain, which varies in length and structure depending on whether it belongs to vitamin D_2_ (nine carbon atoms) or D_3_ (eight carbon atoms) (Fig. 1). In vitamin D_2_, there is an additional methyl group at C24 and a double bond between C22 and C23 (Sirajudeen et al. 2019).

The A-ring contains one hydroxyl group at carbon C3 atom (vitamin D), which may be accompanied by another one at C25 in the aliphatic side chain linked to ring D (giving rise to 25-hydroxy-vitamin D) and a third one at C1α in ring A (giving rise to calcitriol or 1α,25-dihydroxy-vitamin D) (Sirajudeen et al. 2019). These last versions of vitamin D, which contain the C25 and C1α hydroxylations, are the active forms (Jones et al. 2018). The first hydroxylation step, which produces 25-hydroxy-vitamin D, takes place in the liver (Fig. 1) through the action of the enzyme 25-hydroxylase (also called CYP2R1), therefore converting vitamins D_2_ and D_3_ into 25-hydroxy vitamin D_2_ (ergocalcidiol) and 25-hydroxy vitamin D_3_ (calcidiol), respectively. The second and final hydroxylation step takes place in the kidneys (Fig. 1) through the action of the enzyme 1α-hydroxylase (also called CYP27B1), which generates the active forms of vitamin D_2_ and D_3_ (1α,25-dyhydroxy-vitamin D_2_ (ercalcitriol)) and 1α,25-dyhydroxy-vitamin D_3_ (calcitriol). About 85 to 90% of the bioactive form of vitamin D circulating in plasma is not bioavailable, as it is kept bound to the vitamin D binding protein (VDBP), while 10 to 15% is bound to serum albumin, and only about 1% is freely available in plasma, and may be the best biomarker for vitamin D status in patients (Zhu et al. 2022). Last, the kidney hydroxylase, CYP24A1, carries out the formation of 24,25-dihydroxy-vitamin D_3_, which is an inactive catabolic derivative (Borel et al. 2015; Jones et al. 2018; Sirajudeen et al. 2019).

The chemical synthesis of vitamin D in the pharmaceutical industry is a multistep process that demands exacting precision and optimization at each stage to yield the active pharmaceutical ingredient (API) with high purity and efficacy (López-Pérez et al. 2017). The initial step in the synthesis of vitamin D_2_ or ergocalciferol involves the ultraviolet (UV) irradiation of ergosterol, a sterol found in fungi. This photochemical reaction is facilitated by the presence of a suitable solvent system, typically an ethanol and ether mixture, to produce previtamin D_2_. Subsequent thermal isomerization yields vitamin D_2_. However, the UV irradiation process must be carefully controlled to prevent side reactions, such as the formation of lumisterol and tachysterol, which can compromise the yield and purity of the final product (Lythgoe et al. 1978).

For the synthesis of vitamin D_3_ or cholecalciferol, 7-dehydrocholesterol extracted from lanolin undergoes a similar UV irradiation process. The reaction is typically conducted in an inert atmosphere to avoid oxidation and is carried out at low temperatures to enhance the selectivity towards previtamin D_3_. Afterward, previtamin D_3_ thermally isomerizes to vitamin D_3_, which is then purified through various chromatographic techniques to achieve the desired pharmaceutical-grade standards (Lythgoe et al. 1978). The biosynthesis of active vitamin D metabolites, such as calcitriol, involves additional hydroxylation steps. These reactions are catalyzed by cytochrome P450 enzymes, which introduce hydroxyl groups at specific positions on the vitamin D molecule. For instance, the production of calcitriol necessitates hydroxylation at the 1αposition, followed by 25-hydroxylation. The use of microbial fermentation or cell-free systems has been explored to enhance the specificity and yield of these hydroxylation reactions, which are otherwise challenging due to the need for regioselectivity, and the potential for multiple hydroxylation sites on the sterol backbone.

Advances in chemical synthesis have also led to the development of prodrugs and analogs of vitamin D, designed to overcome pharmacokinetic challenges, such as poor bioavailability and reduced stability (Fernández and Ferrero 2020; Kawagoe et al. 2021; López-Pérez et al. 2018). These synthetic analogs often incorporate modifications to the side chain or the A-ring of the molecule, which can enhance their activity or target specific pathways. For example, alfacalcidol is a prodrug that is converted in vivo to calcitriol, circumventing the need for renal hydroxylation, which can be beneficial in patients with renal impairment (Li et al. 2022). Several strategies can be used to enhance the stability of vitamin D APIs during synthesis and storage. These include the stabilization of the molecule with antioxidants such as ascorbic acid, tocopherols, or even *trans*-resveratrol (Díaz-Ruiz et al. 2021), as well as the development of formulations that protect the molecule from light, heat, and moisture. Solid-state characterization techniques, including X-ray crystallography and differential scanning calorimetry, are utilized to ensure the correct polymorphic form of the vitamin D API, which can significantly influence its stability and bioavailability.

In conclusion, the chemical synthesis of vitamin D and its analogs in the pharmaceutical industry is a complex process that requires rigorous control of reaction conditions, the application of advanced synthetic techniques, and meticulous attention to the stabilization and purification of the final product to meet the stringent requirements for pharmaceutical applications.

## Microbial bioconversions of vitamin D

The most archetypal example of bioconversion catalyzed by a bacterial CYP_450_ at the industrial level is the 6-hydroxylation of compound ML-236BNa produced by *Penicillium* to generate pravastatin. This hydroxylation reaction is performed at the industrial level by a soluble CYP_450_ (CYP105A3, P450sca) from *Streptomyces carbophilus* (Sakaki et al. 2011), and CYP_450s_ are the main enzymes used in bioconversion. The *Streptomyces* genus, ubiquitous high G + C Gram-positive soil-dwelling bacteria, is one of the most prolific producers of natural products and it is widely used as a biofactory in the production of several drugs as well as the functionalization of other chemical scaffolds of pharmacological interest (Barbuto Ferraiuolo et al. 2021). The presence of CYP_450s_ in the *Streptomyces* genus accounts for 0.2 to 0.4% of all coding sequences, reflecting the extraordinary biosynthetic potential of these soil microorganisms. More than two-thirds of the characterized CYP_450s_ from *Streptomyces* catalyze hydroxylations which are commonly known as monooxygenases because they only introduce to the substrate one oxygen atom from molecular oxygen, while the other is reduced to water (Rudolf et al. 2017). Given their high catalytic diversity and regioselectivity, they open a new door and represent an efficient alternative to chemical synthesis for the production of different fine chemicals or pharmaceuticals.

In the specific case of vitamin D_3_, bioconversion is a highly attractive field since the chemical synthesis of 1α,25-(OH)_2_-D_3_ from cholesterol requires more than 20 enzymatic steps with complex protections and low production efficiency, C1α-hydroxylation being the most limiting step. Indeed, the classical photochemical ring-opening method of Δ5,7-steroidal dienes yields less than 1% efficiency (Wang et al. 2022). For this reason, there is a considerable amount of research focused on the biology of microorganisms to produce vitamin D_3_ and their analogs via highly regio- and stereoselective processes which offer significant potential for sustainable methods of vitamin D_3_ and derivatives’ biosynthesis (Kang et al. 2014; Pandey and Malik 2019).

Two common bottlenecks in bacterial bioconversion studies of vitamin D are its solubility/cell permeability and the required redox activity associated with CYP_450s_. To solve the first problem, it is common to use cyclodextrins to improve the conversion of the poorly soluble vitamin D, as well as add nisin to generate pores and increase the accessibility of this vitamin within cells (Imoto et al. 2011; Schmitz et al. 2021). Regarding redox partners, one advantage of *Streptomyces* bacterial CYP_450s_ that justifies this type of host is the inherent flexibility of CYP_450s_ to accept redox proteins from other *Streptomyces* species, other bacterial genera, and even eukaryotes (Rudolf et al. 2017; Wang et al. 2022).

In the 1990s, Sasaki et al. massively screened more than 400 species of actinomycetes for their potential to bioconvert vitamin D_3_. Among them, *S. sclerotialus* and *S. roseoporus* were able to convert 25-(OH)-D_3_ and 1α-(OH)-D_3_, respectively, to 1α,25-(OH)_2_-D_3_; however, conversion of vitamin D_3_ to 25-(OH)-D_3_ was not observed in any case. The enzymes in charge were CYP_450s_ (Sasaki et al. 1991, 1992), and the gene coding for 25-hydroxylase of vitamin D_3_ (CYP105A2) from *Pseudonocardia autotrophica* was cloned and heterologously expressed in *S. lividans* (Kawauchi et al. 1994). By definition, bacterial CYP_450s_ are soluble enzymes and require coupled electron transport chains such as ferredoxin and NADPH-dependent ferredoxin reductase; however, the authors were able to see that for heterologous expression of CYP105A2 in *S. lividans* the native ferredoxin-ferredoxin reductase system of this host was able to couple with exogenous CYP450 (Kawauchi et al. 1994) (Table 1).

**Table 1:** List of CYP450s identified in bacteria whose activity has been verified by heterologous expression

Species	CYP_450_	Protein ID	Activity	Cellular location	Heterologous expression host	Coexpression of redox partners	Reference
*Pseudonocardia autotrophica*	CYP105A2	2019246A	Vitamin D_3_ 25-hydroxylase	Cytoplasm	*Streptomyces lividans* TK24 (*str-6*, SLP2^−^, SLP3^−^)	No	Kawauchi et al. 1994
*Pseudonocardia autotrophica*	CYP107 (Vdh)	BAH58688.1	Vitamin D_3_ 25-hydroxylase 25-OH-D_3_ 1α-hydroxylase	Cytoplasm	*Rhodococcus erythropolis* JCM3201	Yes	Fujii et al. 2009
*Streptomyces griseolus*	CYP105A1	P18326.2	Vitamin D_3_ 25-hydroxylase 25-OH-D_3_ 1α-hydroxylase	Cytoplasm	*Streptomyces lividans* TK23 (*spc-1*, SLP2^−^, SLP3^−^)	Yes/no	Hayashi et al. 2010
*Sebekia benihana*	CYP-sb21	6M4S_A	Vitamin D_3_ 25-hydroxylase	Cytoplasm	*Streptomyces coelicolor* M145	No	Bhan et al. 2010
*Bacillus lehensis*	CYP107CB2	AIC83164.1	Vitamin D_3_ 25-hydroxylase	Cytoplasm	*E. coli*	Yes	Ang et al. 2018
*Bacillus megaterium*	CYP109E1	WP_013084555.1	Vitamin D_3_ 24-hydroxylase Vitamin D_3_ 25-hydroxylase	Cytoplasm	*E. coli*	Yes	Abdulmughni et al. 2017

Later, Fujii et al. also described in *P. autotrophica* a hydroxylase CYP_450_ belonging to the CYP107 (Vdh) family capable of transforming vitamin D_3_ into 1α,25-(OH)_2_-D_3_ via 25-(OH)-D_3 _(Kang et al. 2006). This enzyme is widely used industrially and it also catalyzes non-specific hydroxylation at position C26. Heterologous expression of this gene was performed in *Rhodococcus erythropolis*, and it was seen that the hydroxylase activity was low, whereas when it was co-expressed with the redox proteins ThcC and ThcD the activity was increased sixfold. Thus, it seemed that, for this host, redox partners are important, despite the fact that endogenous ones function at a lower rate (Fujii et al. 2009) (Table 1).

In 2010, it was observed that CYP105A1 hydroxylase from *S. griseolus* was able to convert vitamin D_3_ to 1α,25-(OH)_2_-D_3_ via 25-(OH)-D_3_ (Hayashi et al. 2010). In addition, it also had C26 hydroxylation activity, generating a derivative with enhanced antiproliferative activity. The CYP105A1 gene of *S. griseolus* is cotranscribed together with the ferredoxin Fdx1, and although the ferredoxin reductase (Fdr) gene was not found in the vicinity of the CYP105A1 and Fdx1 genes, it appears that the CYP105A1 gene forms an electron transport chain together with Fdr and Fdx1. Heterologous expression of the gene together with Fdx and Fdr was achieved in *S. lividans* under the control of the P_tipA_ promoter (induction by thiostreptone). However, the authors could not confirm the expression of the Fdx and Fdr genes, and the constructs lacking Fdr showed identical bioconversion levels to the constructs with all three genes, suggesting once again that endogenous Fdrs from *S. livindans* operate as electron donors, as had been observed with heterologous expression of CYP105A2 (Hayashi et al. 2010). CYP105A1 hydroxylase exhibits 55% identity at the amino acid level with CYP105A2 from *P. autotrophica*, but there is a clear difference in its catalytic activity, with CYP105A1 hydroxylating at two positions (C1 and C25) and CYP105A2 only at C25 (Wang et al. 2022) (Table 1). The CYP-sb21 hydroxylase from the rare actinomycete *Sebekia benihana*, which belongs to the CYP107 family, generated 25-OH-D_3_ and 1α,25-OH-D_3_ in crude cultures. Its heterologous expression was achieved in *S. coelicolor* under the P_tipA_ promoter without redox partners, but in this case only 25-OH-D_3_ formation was observed (Bhan et al. 2010) (Table 1). In summary, some hydroxylases such as CYP105A1 and CYP107 can hydroxylate at both the C25 position of vitamin D_3_ and the C1α position of 25-(OH)-D_3_ (Table 1).

The ability of the actinomycete *Kutzneria albida* to generate different mono- and dihydroxylated versions of vitamin D_2_ and D_3_ has recently been described (Schmitz et al. 2021). *K. albida* is a minor branch of the *Pseudonocardiaceae* family, so it is related to *P. autotrophica* which is widely known for its ability to catalyze vitamin D hydroxylations (Fujii et al. 2009; Kawauchi et al. 1994). Although its genome was sequenced revealing the presence of 50 CYP_450s_, many of them belonging to the CYP107 family, the gene responsible for the transformation was neither identified nor cloned.

Apart from actinomycetes, different bacterial species from the genus *Bacillus* have been used for biotransformations of vitamin D. In *Bacillus lehensis* G1, the cytosol-soluble cytochrome P_450_ CYP107CB2 is able to carry out the hydroxylation at C25 of both substrates, vitamin D_3_ (preferred substrate) and 1α-hydroxy-vitamin D_3_, giving rise to 25-hydroxycholecalciferol and calcitriol, respectively. This enzyme has been successfully overexpressed in *Escherichia coli* for studying these biotransformations (Ang et al. 2018). As another species from this genus, *B. megaterium* MS941 produces the enzyme CYP109E1, which is used as substrate vitamin D_3_, giving rise to 24-hydroxycholecalciferol, 25-hydroxycholecalciferol and 24,25-dihydroxycholecalciferol (a derivative from the intermediate 24-hydroxycholecalciferol) (Abdulmughni et al. 2017). In conclusion, here we summarized the critical role of microbial bioconversions in vitamin D_3_ synthesis, particularly through the use of bacterial cytochrome P_450_ enzymes. These enzymes, mainly from actinomycete species, demonstrate remarkable specificity and efficiency in hydroxylating vitamin D_3_, producing various bioactive forms. Advances in heterologous expression and the use of redox partners have significantly enhanced these bioconversion processes. This innovative approach offers a sustainable and efficient method for producing vitamin D_3_ and its derivatives, addressing challenges in chemical synthesis and showcasing the potential of microbial systems in pharmaceutical development.

## Data Availability

This review paper does not contain associated laboratory data.
